# Supplementary material for: Very-Long-Chain Acyl-CoA Dehydrogenase Deficiency: Family Impact and Perspectives
Source: Int J Neonatal Screen. 2023 Oct 6;9(4):53. doi: 10.3390/ijns9040053 (PMC10594473; doi:10.3390/ijns9040053)
Supplement: Supplementary file 1 [file IJNS-09-00053-s001.zip › IJNS-2483066-supplementary.pdf]

Supplementary materials

Table S1. Caregiver survey, featuring questions and answer choices.

Question	Answer Choices
1. How old are you (parent)?	
2. What is your marital status?	Single, never married Married or domestic partnership Widowed Divorced Separated
3. How many children do you have?	1 2 3 4 5 >5
4. How many children do you have that are affected with VLCADD?	1 2 3 4 5 >5
5. My child's/children/s VLCADD is difficult to manage	Strongly Disagree (Not difficult at all)<br>Disagree<br>Neither Agree nor Disagree<br>Agree<br>Strongly Agree (Very difficult)
6. Circle all that apply. VLCADD impacts my family's ability to access care by:	Causing financial constraints<br>Limiting access to proper nutrition<br>Increasing emotional stress<br>Impacting ability to work<br>Does not impact daily life
7. Does insurance cover the care needed for your child's/children's VLCADD?	Yes No Other:
8. What recommendations have been made for your child/children for their condition by their metabolic specialist? Circle all that apply.	a. Avoid fasting<br>b. Increased meal frequency<br>c. Avoid prolonged exercise<br>d. Use a fatty acid supplement (MCT oil, L-carnitine, or other fatty acid supplement)<br>e. Use a carbohydrate supplement<br>f. Follow a low-fat diet<br>g. Follow a high carbohydrate diet<br>h. Multi-vitamin/mineral supplement<br>i. Other:
9. What of the current recommendations are you following for your child's/children's condition?	a. Avoid fasting<br>b. Increased meal frequency<br>c. Avoid prolonged exercise<br>d. Use a fatty acid supplement (MCT oil, L-carnitine, or other fatty acid supplement)<br>e. Use a carbohydrate supplement<br>f. Follow a low-fat diet<br>g. Follow a high carbohydrate diet<br>h. Multi-vitamin/mineral supplement<br>i. Other:
10. How often do you receive services from a metabolic genetics provider?	"As often as recommended by the provider - if they answer this, then how often?<br>Only if my child gets sick/hospitalized<br>Based on nutritional needs<br>When I remember<br>My child does not see a metabolic provider "
11. Does your child's/children's daycare or school have a specific plan for your child's VLCADD?	Yes<br>If yes, is it a 504 plan?[1]

		If no, what is it?<br>No<br>Not Applicable	
12.	Does the school have a dietary plan recommended by the specialist?	Yes	No
13.	Is the child's/children's pediatrician made aware of the VLCADD diagnosis?	Yes	No
14.	Open-ended—Is there anything else you would like to add?		

[1]A 504 plan is a document describing changes or modifications that a student may need in school.
